# Brachial Plexus Injury and Musculocutaneous Nerve Palsy During Prone Positioning in a Patient With COVID-19

**DOI:** 10.7759/cureus.24931

**Published:** 2022-05-11

**Authors:** Tomoo Mano, Shigekazu Fujimura

**Affiliations:** 1 Department of Rehabilitation Medicine, Nara Medical University, Kashihara, JPN

**Keywords:** musculocutaneous nerve, brachial plexus, prone positioning, injury, covid-19

## Abstract

Prone positioning is crucial in the respiratory management of patients with acute respiratory distress syndrome (ARDS) and reduces mortality. However, this may be complicated by compression-related peripheral nerve injury. We report the case of a male in his 80s with obesity admitted to the intensive care unit (ICU) with COVID-19 pneumonia who developed brachial plexus disorder in the right upper extremity and musculocutaneous neuropathy in the left. The patient’s cough, dyspnea, and fatigue did not improve; therefore, he was intubated and placed in the prone position for one week. The patient complained of bilateral upper limb weakness on regaining consciousness. We diagnosed left musculocutaneous nerve palsy and right brachial plexus palsy based on physical findings and needle electromyography (EMG). Physical therapy was initiated, including joint range-of-motion exercises focused on preventing contractures in the extremities and active assistive exercises. Motor impairment improved, and the patient was discharged from the rehabilitation center.

## Introduction

Prone ventilation, an established component of the management of acute respiratory distress syndrome (ARDS), is recommended in cases of moderate to severe ARDS according to the coronavirus disease 2019 (COVID-19) clinical guidelines [1]. Early intervention with prone positioning significantly reduces mortality in patients with severe ARDS; however, it is associated with adverse events such as endotracheal tube obstruction, pressure ulcers, and peripheral nerve injury [2]. Herein, we report a case of bilateral upper extremity paralysis caused by prone positioning to manage COVID-19. This case of a patient, who developed brachial plexus disorder and musculocutaneous neuropathy because of prone ventilation in the intensive care unit (ICU), reveals the risk of peripheral neural injury caused by long-term prone positioning.

## Case presentation

**Figure 1:**
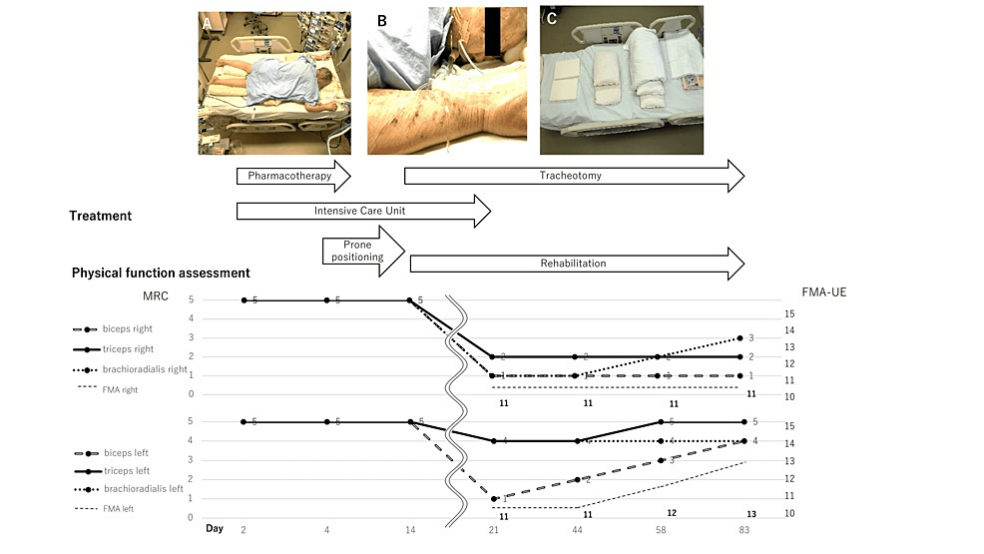
Prone positioning in the ICU, treatment course, and physical function assessment A: Prone positioning. B: Cervical rotation to the right, a position to avoid airway problems and blockage of the intubation tube. C: Cushions used for positioning. The physical function assessment suggested brachial plexus disorder in the right upper extremity and musculocutaneous neuropathy in the left upper extremity. MRC Muscle Scale: Medical Research Council Muscle Scale; FMA-UE: Fugl-Meyer Assessment-Upper Extremity Scale

A male in his 80s with a history of bladder cancer, hypertension, abnormal glucose tolerance, chronic heart disease, and paroxysmal atrial fibrillation visited a local health provider with complaints of high fever (38.4°C), anorexia, and frequent urination. He was admitted to our hospital after a positive result on the rapid antigen test for COVID-19 (Figure 1). He was administered remdesivir from hospitalization day (D)1 and transferred to the ICU for endotracheal intubation and mechanical ventilation on D2 as his respiratory function deteriorated. Prone positioning was applied during D4-D11 under local guidance, with reference to the Society of Critical Care Medicine guidelines [3]. Before performing prone positioning, we placed a pressure-resistant disperser (Purefix, one sheet for Purefix, three cushions) on the lower limbs, ilium, and precordium with the patient in the supine position. Soft decompression pillows were placed under the face, with the position adjusted to eliminate pressure on the eyeballs and ears. Further, the pressure-resistant disperser was positioned to avoid pressure on the abdomen and genital area. The postural parameters included head at 10° extension, neck right rotated at 30° flexion, shoulder at 80° abduction/90° external rotation, and elbow at 90° flexion. Nurses repositioned the patient hourly with attention to the face, shoulders, chest, and groin. Cushions were placed under the head, shoulders, and pelvis at heights of 10, 25, and ~20 cm, respectively, to prevent pressure ulcers, and their positions were adjusted every three hours. Furthermore, body repositioning and rolling had to be minimized because of the patient’s obesity and risk of airway problems and endotracheal tube obstruction. The environment precluded the use of devices for monitoring somatosensory evoked potentials [4]. Tracheostomy was performed on D14. The patient was assessed for rehabilitation on recovering consciousness after induction of anesthesia.

Investigations

General Physical Examination

The general physical characteristics were as follows: blood pressure, 138/86 mmHg; respiratory rate: 23 breaths/minute; temperature, 37.5°C; height, 161 cm; weight, 78 kg; and body mass index, 31.1 kg/m^2^. Moist rales were detected in both lungs. Mild edema was observed in all extremities without evidence of pressure ulcers. Chest radiography revealed a dense infiltrate in the lower lobe of the right lung. The results of laboratory tests performed on D14, D20, and D35 showed no evidence of other peripheral neuropathies, such as inflammation, infection, or collagen disease.

Neurological Findings (D14)

The level of consciousness on the first assessment was rated as follows: eye-opening (E), 4; verbal response (V), tracheal intubation; and motor response (M), 6 (Glasgow Coma Scale). Both deltoid muscles were weak, and the patient could not pronate the left arm. The left triceps brachii was also weak. The biceps brachii were weak bilaterally, and coracobrachialis function was weak on the left and nearly normal on the right. The Medical Research Council (MRC) Muscle Scale scores for the upper extremities were as follows: deltoid, 2/2; triceps, 2/4; biceps, 0/1; supinator, 2/4; and brachioradialis, 1/4. The patient could not either flex the elbow or pronate the right forearm. Function was impaired in the right-hand finger muscles innervated by the median nerve but retained in those innervated by the ulnar nerve. Motor paralysis was not evident in the lower extremities. Superficial sensation was reduced in the ulnar forearm and right-hand fingers, suggesting a right musculocutaneous lesion. Tendon reflexes were reduced in the upper extremities but were normal in the lower extremities. Neither Wartenberg’s sign nor any other pathological reflexes, such as palmomental, Babinski, or Chaddock’s reflex, were observed. The patient scored 11 points on the Fugl-Meyer Assessment-Upper Extremity Scale (FMA-UE).

Treatment course

Rehabilitation therapy was started on D14 to strengthen the muscles and prevent disuse syndrome. It included positioning, respiratory physiotherapy, joint range-of-motion exercises focused on preventing contractures in the extremities, automatic generated exercises, and active assistive exercises. Occupational therapy was added on D21. Subsequently, neuromuscular re-education was performed in tandem with electric muscle stimulation following the patient’s relocation to the general isolation ward.

Needle electromyography and nerve conduction study

The patient’s neurological symptoms showed no improvement, even with rehabilitation therapy. We performed needle electromyography (EMG) on D45 using MEM-8301 Neuropack n1 (Nihon Kohden Corporation, Tokyo, Japan) to assess the upper extremity muscles. The procedure was delayed because of the required permission from the hospital review board to transfer the portable EMG device into the ICU. The findings are summarized in Table 1. The patient’s reduced muscle strength complicated the evaluation of interference patterns. We performed a nerve conduction study on D40 and D70. Compound motor action potential-stimulated wrists and elbows were within the normal range on both days. Sensory nerve action potential (SNAP) was within the normal range on D40, but the amplitude of SNAP in the left median nerve and radial nerve and right median nerve was low on D75.

**Table 1:** EMG summary IA: insertional activity; Fib: fibrillation potential; PSW: positive sharp wave; Fasc: fasciculation; MUAP: motor unit action potential; Amp: amplitude; Dur: duration; PPP: polyphasic potential

			Spontaneous				MUAP			Recruitment
Muscle	Nerve		IA	Fib	PSW	Fasc	Amp	Dur	PPP	Pattern
R. Deltoid	Axillary nerve		N	一	一	None	N	N	1+	N
R. Biceps	musculocutaneous nerve		N	十	十	None	1+	1+	1+	dec
R. Triceps	Radial nerve		N	一	一	None	N	N	N	N
R. Extensor digitorum muscle	Radial nerve		N	一	一	None	N	N	N	N
R. First dorsal interosseous	Ulnar nerve		N	一	一	None	N	N	N	N
L. Deltoid	Axillary nerve		N	十	十	None	1+	N	1+	dec
L. Biceps	Musculocutaneous nerve		N	十	十	None	N	N	1+	dec
L. Triceps	Radial nerve		N	十	十	None	1+	N	1+	dec
L. Extensor digitorum muscle	Radial nerve		N	十	十	None	1+	N	1+	dec
L. First dorsal interosseous	Ulnar nerve		N	一	一	None	N	N	N	N

Outcome and follow-up

The sensory disturbances showed no improvement or worsening. However, the patient had muscle contraction in his left biceps by D58, flexion in his elbows by D67, and contraction in his right biceps by D70. The cervical spine and chest magnetic resonance imaging excluded radiculopathies and brachial plexus neuritis. The MRC ratings (triceps, 2/5; biceps, 1/4; brachioradialis, 1/4) and FMA-UE score (13 points) improved. No abnormalities were found in cervical spine magnetic resonance imaging. The patient was discharged on D83.

## Discussion

Peripheral neuropathy due to COVID-19 has been reported [5]; however, there are no reports of peripheral neuropathy involving both upper limbs. Peripheral neuropathy in the upper extremities due to prone ventilation in COVID-19 management can be attributed to compression and traction involving the brachial plexus and ulnar, radial, posterior interosseous, and musculocutaneous nerves [5]. Localized impairment of nerve conduction generally has good outcomes when only the myelin sheath is affected; however, outcomes worsen with axonal damage due to long-term compression/traction. Based on this patient’s course, brachial plexus neuropathy could be due to axonal damage. The neuropathy could be due to nutritional demands, neuroinflammatory changes, critical illness polyneuromyopathy, or peripheral nerve trauma. We excluded the conditions in the differential diagnoses with limited initial examinations [6].

Sites vulnerable to compressive neuropathy in the prone position include the medial and lateral epicondyles of the humerus, humeral head, and ulna [7]. Prone positioning can cause signs of severe injury to the brachial plexus after just one hour of compression [4]. Obesity, diabetes, and older age are significant predictors of peripheral neuropathy during prone positioning [7]. Traction forces, in addition to compression, are also highly involved in peripheral neuropathy, given the absence of skin damage in many such cases, including ours. The brachial plexus is susceptible to compression and extension because of its long course and adjacency to the clavicle and humeral head [8]. Injuries frequently occur in the prone position because of traction when the shoulder is hyper-abducted [4]. The left brachial plexus in our patient might have been damaged near the lateral nerve fascicle, overstretched by shoulder abduction due to the external rotation and posterior displacement [9]. Conversely, the musculocutaneous nerve is more susceptible to injury during excessive movement from the shoulder to the elbow, for example, when the coracobrachialis is extended during abduction or external rotation of the upper extremities or when it is contracted, and traction is applied to flex the head to the contralateral side. Our patient’s head was not flexed during prone ventilation. However, his joint angles were similar to those at which musculoskeletal neuropathy has been reported in the past [10], suggesting that the injury occurred distal to the humeral head.

Our patient was overweight (body mass index: 31.1 kg/m^2^). Obesity is a major risk factor for poor respiratory function in COVID-19 [11], and many such cases require prone positioning. The reason underlying the frequent occurrence of peripheral neuropathy in overweight, prone-positioned patients with COVID-19, besides stronger compressive and traction forces due to greater body mass, is the likelihood of more severe breathing difficulties requiring prone ventilation. Bodyweight has been incorporated into the guidelines for body pressure distributions and peripheral neuropathy in the supine position, but not into the reference values for body pressure in the prone position.

In our case, bilateral neuropathy resulted from the lack of experience with repositioning and relieving compression in overweight patients under prone ventilation. Neuropathy was inferred based on physical findings and results of needle EMG that was permitted inside the COVID-19 ward. Because the patient was transferred to another center after his discharge from isolation, we were unable to perform nerve conduction studies to corroborate.

## Conclusions

We intend to draw attention to the risk of peripheral nerve injury caused by prolonged prone positioning. Peripheral nerve injury is becoming a significant issue during the COVID-19 pandemic and is more severe in obese patients. The long course of the brachial plexus, its firm fixation to the prevertebral and axillary fascia, and its close association with the clavicle, first rib, and humerus underlie its vulnerability. Compression and traction during prone positioning results in periaxonal edema, ischemia of the vasa nervorum, demyelination, and, in severe cases, axonal degeneration. Careful positioning and extra padding in areas where the peripheral nerves may be exposed to pressure are recommended.
